# Typological thinking in human genomics research contributes to the production and prominence of scientific racism

**DOI:** 10.3389/fgene.2024.1345631

**Published:** 2024-02-19

**Authors:** Kevin A. Bird, Jedidiah Carlson

**Affiliations:** ^1^ Department of Plant Sciences, University of California, Davis, CA, United States; ^2^ Department of Integrative Biology and Department of Population Health, University of Texas, Austin, TX, United States

**Keywords:** genomics, race, scientific racism, typological thinking, open science, genetic ancestry

## Abstract

Public genomic datasets like the 1000 Genomes project (1KGP), Human Genome Diversity Project (HGDP), and the Adolescent Brain Cognitive Development (ABCD) study are valuable public resources that facilitate scientific advancements in biology and enhance the scientific and economic impact of federally funded research projects. Regrettably, these datasets have often been developed and studied in ways that propagate outdated racialized and typological thinking, leading to fallacious reasoning among some readers that social and health disparities among the so-called races are due in part to innate biological differences between them. We highlight how this framing has set the stage for the racist exploitation of these datasets in two ways: First, we discuss the use of public biomedical datasets in studies that claim support for innate genetic differences in intelligence and other social outcomes between the groups identified as races. We further highlight recent instances of this which involve unauthorized access, use, and dissemination of public datasets. Second, we discuss the *memification,* use of simple figures meant for quick dissemination among lay audiences, of population genetic data to argue for a biological basis for purported human racial groups. We close with recommendations for scientists, to preempt the exploitation and misuse of their data, and for funding agencies, to better enforce violations of data use agreements.

## Introduction

Genetics, evolutionary biology, and biomedical research have been revolutionized by the advent of public genomic datasets like the 1000 Genomes Project (1KGP), the Human Genome Diversity Project (HGDP), and the numerous datasets hosted on various data archives by the National Institutes of Health (NIH), including the database of Genotypes and Phenotypes (dbGaP), the National Institute of Mental Health Data Archive (NDA), and the Sequence Read Archive (SRA). These resources have empowered researchers worldwide. Access to vast troves of genetic information has been democratized, collaborative efforts that were once inconceivable are possible, and breakthroughs in understanding the intricacies of human evolution and disease etiologies have been drastically accelerated.

Coinciding with these developments has been a resurgence in the volume and prominence of scientific racism (Saini, 2019), defined by Bird, Jackson, and Winston (In press) as “the use of scientific concepts and data to create and justify an enduring, biologically-based racial hierarchy.” The scientific racism movement manifests in several forms, from extremist online social media communities to academic literature (as described in Panofsky et al., 2021; Bird et al., 2023), to popular press books like Nicholas Wade’s *A Troublesome Inheritance: Genes, Race and Human History* (Wade, 2015) or Charles Murray’s *Human Diversity: The Biology of Gender, Race, and Class* (Murray, 2020). This movement’s presence and use of mainstream scientific research is of concern due to the frequency with which participants overlap with white supremacist groups, hold anti-democratic and anti-egalitarian sentiment, and, in the most extreme cases, carry out or contribute to violent terrorist attacks (Bird et al., 2023; Carlson et al., 2022).

Previous work has shed light on how certain open science practices have been abused or co-opted by scientific racists; for example, Carlson and Harris (2020) document the frequent prominence of white supremacist communities in social media discussions of scientific preprints on bioRxiv (Carlson and Harris, 2020). Panofsky, Dasgupta, and Iturriaga (2021) provide a detailed exploration of how principles of the open science movement (e.g., Fecher and Friesike, 2014) were exploited by the *OpenPsych* journals to provide a venue for, and legitimacy to, the publishing of scientific racism. However, less attention has been paid to the role of public genomic datasets, and practices in mainstream research when constructing and analyzing such datasets, in facilitating co-option by the scientific racism community. We specifically focus on how the failure of human genetics research to fully separate from essentialist and typological conceptions of racial categories (e.g., that the groups identified as races are largely homogenous, discrete, and reflect fundamental biological divisions with discrete morphology and psychology; see Dobzhansky, 1950) has primed mainstream research to be misappropriated by scientific racists (Table 1).

**TABLE 1:** Summary of research practices, harms, and proposed solutions.

Problematic research practice	Potential harms	Proposed solution
Overemphasis on genetic determinants of racial health disparities	- Contributes to genetic essentialism	- Authors should assess whether emphasizing genetic determinants is scientifically justified and how that relates to the conceptualization of genetic ancestry used in the study
	- Undermines research on social determinants of health or role of racism in health disparities	- Journal editors and reviewers should be equipped to critically and constructively address work that defaults to an emphasis on genetic determinants of health without clear rationale and justification
		- Funding agencies should be encouraged to prioritize work that engages with both environmental and genetic determinants
Causal interpretation of correlative genetic research	- Obscures impact of known environmental confounding in polygenic scores calculated from genome-wide association studies at the population level or correlations between genetic ancestry and a trait	- Authors should be clear about what causal parameters they are attempting to estimate and how those estimates are affected by environmental confounding. Interpretations or conclusions should be appropriately tempered
	- Stigmatizes marginalized groups	- Editors and reviewers at journals and funding agencies should assess submitted manuscripts and grant proposals for clarity and forthrightness on this matter
	- Contributes to genetic essentialism	
Conflation of genetic ancestry and race and use of categorical genetic ancestry groupings	- Contributes to genetic essentialism and typological thinking	- Researchers should avoid categorical genetic ancestry labels as much as possible and replace them with continuous representations of variation
	- Frequently provides oversimplified representation of human evolution and genetic variation	- Researchers should clearly justify the particular representation of genetic ancestry used in a study
		- Researchers should prioritize the development of novel methods and visualizations that embrace the continuous patterning of human genetic variation
Unauthorized access of datasets or dishonesty in data use requests	- Breach of participant privacy and data security	- Improved vetting of data use requests
	- Violation of participants’ informed consent	- Develop appropriate sanctions and punitive measures in cases of egregious and intentional unauthorized use and sharing of federally maintained datasets, even beyond datasets and investigators funded by federal agencies
	- Reduced trust in biomedical institutions	- Consider expanding the purview of the US Office of Research Integrity to cover cases of egregious data misuse

We document two ways in which typological thinking in human genomics research contributes to downstream production and prominence of scientific racism. First, we show how categorical descriptors of genetic ancestry (which includes continental, geographic, political territorial, ethnic, and religious labels), and a conflation of genetic ancestry with folk racial categories like white, Black, and Asian (National Academies of Sciences, Engineering, and Medicine, 2023), in publicly available global genetic diversity and biomedical datasets are re-analyzed in racial hereditarian research to offer support to their hypotheses about the genetic inferiority of Black and African peoples. We further highlight how the latter case has recently involved the unauthorized access and re-use of NIH biomedical datasets and how the scientific community might address this problem. Second, we show how typological thinking influences the sampling, labeling of individuals, and interpretation of results included in catalogs of human genetic variation in ways that inadvertently reinforce racial hereditarian interpretations. We connect this to the co-option of data visualizations like principal component analysis (PCA) or STRUCTURE plots into far-right memes meant to persuade social media users of the “biological reality of race.”

## Misusing public datasets for racial hereditarian research

Panofsky et al., 2021 provide one of the few discursive analyses of white supremacist engagement with scientific research. One of the main engagements they note is the formation of a “racial hereditarian counterscience,” wherein ersatz researchers aim to conduct and publish studies on racial differences free from the perceived dogma of mainstream scientific consensus. The fundamental error in this research is that it operates from a framework of genetic essentialism, where racial groups (often viewed through the lens of contemporary US census categories like “white,” “Black/African American,” and “Asian”), are taken to be typological (e.g., largely genetically homogenous and discrete) and that racial differences in behavior, psychology, and physiology are attributed to these discrete racial genetic differences (see Dar-Nimrod and Heine, 2011; Donovan, 2022). This framework lends itself to strong conflations of racial categories and genetic ancestry and to faulty causal reasoning from confounded genetic results (as discussed in Bird et al., 2023).

Given their frequent lack of funding and/or affiliation with recognized research institutes, these researchers heavily rely on unrestricted publicly available datasets and summary statistics to conduct their research. For example, it is standard practice in genome-wide association studies (GWAS) to publish the genomic coordinates (or a unique identifier, typically the rsID as used in dbSNP), estimated effect sizes, and *p*-values for all genetic variants found to have statistically significant associations with the trait (s) being studied (data from the majority of published GWAS have been compiled and standardized in the GWAS Catalog, a publicly available resource). A common analysis of racial hereditarian researchers is to then cross-reference a set of trait-associated variants in public datasets such as 1KGP or the Genome Aggregation Database (gnomAD) which provide allele frequency estimates of each genetic variant in different human populations. Although several sources of environmental confounding in GWAS done at the population-scale are now known and considered to invalidate any causal inference (See Bird et al., 2023), these differences are presented by racial hereditarian researchers as evidence that phenotypic differences among populations are caused by innate genetic differences. An example of this line of reasoning is advocated by Charles Murray (2020) in the popular press book *Human Diversity*, which shows that SNPs from the GWAS catalog associated with various cognitive, behavioral, and personality traits differ in estimated allele frequency among 1KGP populations. Murray concludes that these population-level genetic differences are a significant source of average phenotypic differences. Similar to Murray (Piffer 2015; Piffer, 2019; Piffer, 2023), uses genotype frequencies of African and European populations from 1KGP and gnomAD to calculate polygenic scores based on published summary statistics from GWAS of intelligence and educational attainment. Piffer (2015), Piffer (2019), Piffer (2023) also advocates for the position that these derived statistics support the argument that international differences in IQ test scores are primarily caused by evolved genetic differences among populations.

Recently, racial hereditarian researchers have capitalized on the availability of biomedical datasets that include racially diverse sampling, genetic sequence data, and phenotypic measurements of participants. These include the Philadelphia Neuroimaging Cohort (PNC), Pediatric Imaging, Neurocognition, and Genetics Study (PING), and the Adolescent Brain Cognitive Development Study (ABCD). These studies predominantly focus on regressing an estimate of European genetic ancestry from the program Admixture against various phenotypes ranging from scores from cognitive test batteries, income, and neuroimaging data (examples include Lasker et al., 2019; Kirkegaard et al., 2019; Fuerst et al., 2021a; Fuerst et al., 2021b; Fuerst et al., 2021c; Kirkegaard and Fuerst, 2023; Fuerst et al., 2023a; Fuerst et al., 2023b; Hu et al., 2023; Shibaev and Fuerst, 2023). Despite known issues with this admixture regression approach to distinguish whether an ancestry-trait correlation is caused by genetic effects or covarying environmental effects (Schraiber and Edge, 2023) these papers make bold claims about genetic differences explaining a substantial portion of racial differences in intelligence, educational attainment, and parental income among Black and white Americans. Furthermore, researchers involved in curating datasets such as the ABCD study have specifically noted the non-representativeness of the sample and the threat of omitted variables related to social and economic inequality as a reason to avoid making strong conclusions (Simons et al., 2021). These applications are particularly concerning as they can lead to stigmatization of a marginalized group, especially given the history of scientific racism and claims of the intellectual inferiority of Africans and Black Americans. These cases also raise questions about informed consent, given the original studies were designed and study participants were recruited with the understanding that results would be used for medical research. Both issues undermine the trust that citizens, especially those from marginalized communities, have in biomedical researchers and institutions. As these institutions are still attempting to repair relationships with marginalized communities from the plentiful cases of abuse and mistreatment throughout the 20th century, it is even more important to maintain their integrity and prevent misappropriation from racial hereditarian research.

While any public dataset may be seized upon by these researchers, mainstream research often, either intentionally or unintentionally, makes assumptions in line with genetic essentialism that might make it easier to co-opt data for racial hereditarian research and occasionally find purchase in legitimate scientific journals. One is the emphasis biomedical research places on genetic determinants of racial health disparities over social determinants (Kaplan and Fullerton, 2022). Kaplan and Fullerton (2022) argue that such a framework primes the interpretation of race as a meaningful genetic variable and views racial health disparities through a genetic lens, not only reifying race but also hampering the investigation of how racialization and racism produce health disparities (e.g., Ivey Henry et al., 2023). Work operating from a framework of genetic determinants of health disparities also frequently involves strong causal interpretation of environmental correlations with polygenic scores, or of mean group differences in polygenic scores. As Kaplan and Fullerton (2022) document, such interpretations are currently highly fraught. Another issue is that when genetic ancestry is considered separately from self-identified race and ethnicity, it is conceived in terms of discrete categories at the continental level. Both PING (Jernigan et al., 2016) and the ABCD release 3.0 provide ancestry assignments by assigning samples to genetic clusters using programs like STRUCTURE and Admixture and a reference panel from HGDP or 1KGP. Such approaches reinforce typological notions of genetic ancestry and are frequently mapped back onto racial categories by researchers (Fujimura and Rakagopalan, 2011; Fujimura et al., 2014; Wills, 2017). While the racial hereditarian studies discussed calculate their own ancestry components with programs like Admixture, they reference the ABCD’s dataset as justification for such practices (Fuerst, Kirkegaard, and Shibaev, 2023a).

Especially concerning is that several of these research endeavors appear to involve dishonest or unauthorized access and use of public datasets, posing a serious threat to the integrity of biomedical science and the trust between the NIH and NIH-funded investigators, and their research subjects. For example, Lasker et al. (2019) were recently implicated in a case of research misconduct where the NIH concluded that the senior author and principal investigator responsible for requesting the PNC data from DbGaP, Dr. Bryan Pesta, had committed a rash of violations to the Data Use Certification Agreement. The NIH subsequently ordered Pesta to destroy any copies of the dataset by June, 2021, revoked his permission to use any NIH data for any ongoing projects, and banned him from accessing NIH data for 3 years, their strongest sanction against a researcher for misusing dbGaP data in the history of the database (Standifer, 2022).

Despite this punitive action ostensibly nullifying all pre-existing Data Use Certification Agreements held by Dr. Pesta (and, in turn, cutting off the access of his collaborators to said data), Dr. Pesta’s institution, Cleveland State University, concluded that John Fuerst, a graduate student and coauthor of Lasker et al. (2019), had retained an unauthorized copy of the ABCD dataset (Standifer, 2022). Fuerst has since published at least 8 preprints and papers analyzing the ABCD data, with at least 5 other coauthors (Fuerst 2021a; Fuerst et al., 2021b; Fuerst et al., 2021c; Fuerst et al., 2023a; Fuerst et al., 2023b; Hu et al., 2023; Kirkegaard and Fuerst, 2023; Shibaev and Fuerst, 2023). Searching the NIMH Data Archive’s database of approved Data Use Certification (DUC) Agreements, we did not find a single DUC requesting access to the ABCD data that had been granted to Fuerst, nor to any of the coauthors of these recent papers. These findings suggest that, in defiance of the NIH’s sanctions, Fuerst has not only retained an unauthorized copy of the ABCD dataset, but has leveraged it to conduct and publish analyses that were never reviewed by NIH staff for adherence with research subject consent and protection. Given that several of these articles are published outside of mainstream venues and by a group of researchers who lack institutional affiliations (or whose affiliation is outside of the United States), the ability to retract or sanction these researchers through usual mechanisms seems minimal.

## “Memification” of human population genetics research

In their analysis of white supremacist engagement with genetic research, Panofsky et al., 2021 also describe a strategy among alt-right communities of sharing “ready-to-go memes, images, and discursive objects that can be circulated online to spread racial realism and hereditarianism” (Panofsky et al., 2021, p. 6), termed “red-pills.” Commonly featured in these “red-pills” are PCA and STRUCTURE plots of global human genetic datasets (e.g., Rosenberg et al., 2002; Rosenberg et al., 2005; Li et al., 2008; Xing et al., 2010), often decontextualized, edited, and relabelled, which are employed during debates to provide bold, visual support for the genetic distinctiveness of so-called races with a facade of technical sophistication. It is commonly claimed by researchers in genetics and evolutionary biology that Darwin’s theory of evolution and the modern synthesis replaced typological thinking about race and with a populationist interpretation, where the groups identified as races are equivalent to subspecies and represent populations with statistically distinguishable allele frequency patterns, marking a retreat from scientific racism (Gannett, 2001). However, Gannett (2001) argues, the typological/populationist distinction, by still maintaining a race concept, and ignoring that genetic differences are interpreted and situated within particular social and historical contexts, failed to represent a sufficient split from typological thinking. As such, there is no assurance that human genetics research will be non-racist or resistant to the influence of racist social structure (Gannett, 2001). While recent efforts are moving to create a more robust division from typological thinking (e.g., Roseman, 2014; Lewis et al., 2022; National Academies of Sciences, Engineering, and Medicine, 2023), much of 21st century human genetics was insufficiently distinct from typological thinking of the 20th century.

**FIGURE 1:**
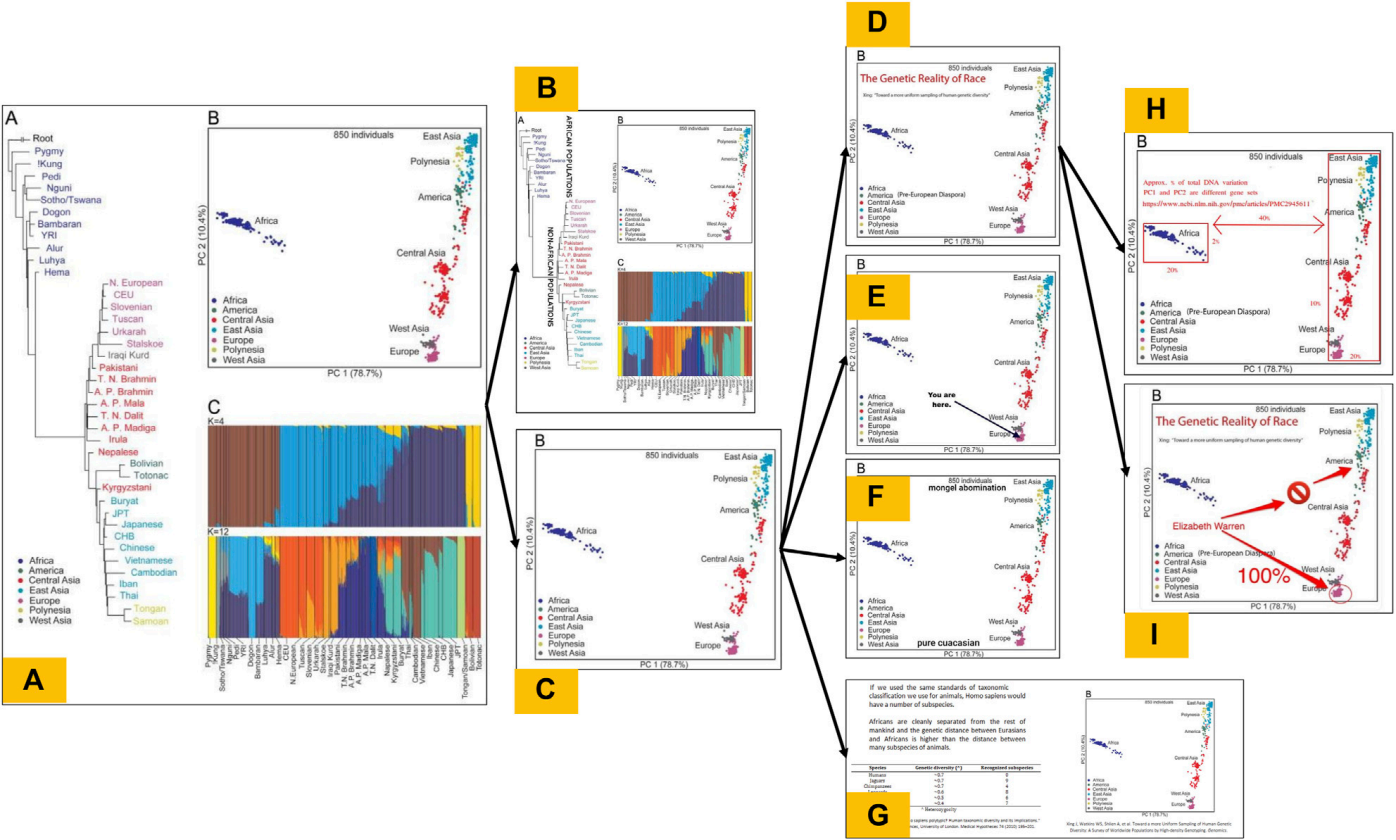
The evolution of a family of race science memes. (A) depicts the original, unmodified Figure 3 published Xing et al., 2010, which contains three panels: an inferred coalescent tree **(A)**, a PCA plot **(B)**, and a STRUCTURE plot **(C)** summarizing the data sampled in the study, and **(B–I)** show various memetic modifications of this figure that we have documented across 4chan and other imageboards popular among far-right audiences. **(B)** Shows a modification of **(A)** in which the full figure is reproduced, but has been modified with additional annotations. **(C)** Shows a modification of **(A)** in which the PCA subpanel has been decontextualized from the other subpanels, but contains no further annotations. **(D–F)** Show secondary modifications of **(C)** in which various textual or graphical annotations have been made. **(G)** Shows a modification of **(C)** in which the PCA subpanel remains unannotated, but it has been combined with a screenshot of a table from another paper into a standalone image. **(H–I)** Show tertiary modifications of **(D)**, which add or remove annotations to convey other interpretations of the figure.

Carlson et al. (2022) identified thousands of posts on the far right website 4chan that involved a meme titled “The Truth About Race” which includes images from several scientific papers, including PCA plots from Li et al., 2008; Xing et al., 2010. The analysis not only revealed posts going as far back as 2016, but that the rate of posting the image has increased over time, and surges in posts often coincided with major political events like the murder of George Floyd, the 2020 election, and the January 6th Capitol insurrection (Carlson et al., 2022). Modified versions of the PCA plot from Xing et al., 2010 (Figure 1) alone are present in over 1,000 posts on 4chan across 12 years and many more across similar far-right imageboard websites. It is also commonly featured in blogs and social media posts from those in the “human biodiversity” movement, a sanitized moniker for this community of scientific racists (Carlson et al., 2022). When these scientific figures are posted, there is little variation in the content whether it is posted on niche, extreme sites like 4chan or mainstream social media sites like Reddit or Twitter (now known as X).

Memes also arise from the integration of multiple public genomic datasets. For example, within weeks of the publishing of a genome-wide association study linking genetic variation to differences in educational attainment (Lee et al., 2018), a post on 4chan was made that cross-referenced the top reported SNPs from Lee et al. (2018) with allele frequencies of African and European populations from the 1KGP dataset. The author of the 4chan post then selected the education-associated SNPs with the greatest frequency difference between populations to show Africans are genetically predisposed to lower intelligence. Within the original thread, the meme was workshopped and modified to more effectively spread this distorted message across online communities (Carlson et al., 2022). Carlson et al. (2022) further documented the rapid proliferation of this meme across social media, including over 5,000 posts on 4chan and many across Reddit, Twitter, and Quora, which culminated in the white supremacist responsible for murdering 10 people in a Buffalo, NY grocery store including the image in his screed with the description “The latest findings on genetics and intelligence show that biological factors contribute to the gap in intelligence between European and African populations” (Carlson et al., 2022; Carlson et al., 2022).

One reason why white supremacist communities have been so easily able to co-opt visualizations from mainstream scientific publications likely relates to the sampling decisions made in the construction of the datasets. In the construction of the HGDP, preference was given to “primitive groups” and “isolates of historical interest” who putatively represent “pure” (i.e., “unadmixed”) samples and thus maximize the extent of human genetic diversity in the sample (Brodwin, 2005; Saini, 2019). Both HGDP and 1KGP also have scarce sampling of African populations despite the fact the continent harbors the greatest amount of genetic diversity among human populations. These choices contribute to the clean separation of clusters in PCA plots and the correspondence between STRUCTURE results with continental populations. Carlson et al. (2022) highlight the impact of these sampling schemes. While HGDP and 1KGP give populations that roughly correspond to major continental groups when K = 7, a broader sample of African populations results in 5 out of 7 clusters corresponding to African subpopulations. Similarly, the use of the BioMe dataset based on samples from New York City results in a PCA plot where the apparent separate continental clusters from HGDP and 1KGP are not distinct but blur together as a continuum of human genetic variation (Lewis et al., 2022). This underpins how the neatly separated, continent-associated clusters from PCA and STRUCTURE plots are, at least partially, an artifact of sampling schemes.

Visualization and textual framing decisions may also contribute to the ease of misinterpretation and misappropriation of population genetic analyses (see discussions in Wills, 2017; Biddanda et al., 2020; Novembre, 2022; Carlson and Harris, 2022). For instance, the paper describing the Phase 3 release of the 1000 Genomes Project (1000 Genomes Consortium, 2015) tends to color figures according to major continental regions of the samples, even when analyses do not support such clusters (e.g., number of polymorphic sites or number of rare variants). Rhetorical analysis of Rosenberg et al. (2002) argues that the framing of results in the abstract and text (such as emphasizing the correspondence between K = 5 clusters and major continental regions)—though presented carefully, impartially, and in a manner appropriate for a primary audience of other researchers—inadvertently predisposed the article to racial hereditarian interpretations (Wills, 2017).

## Recommendations

Addressing these pervasive modes of appropriation will require changes to community and regulatory norms. First, researchers ought to continue embracing efforts to abandon categorical, and especially continental, ancestry labels and replace them with continuous descriptors of genetic similarity that specify the geographic and temporal context (Lewis et al., 2022; National Academies of Sciences, Engineering, and Medicine, 2023). Fortunately, such a change was implemented in the ABCD 4.0 release (Fan et al., 2023; Maes et al., 2023) and in discussions on how to stratify allele frequencies for assessment of variant pathogenicity in clinical settings (Nelson et al., 2022). There is still a need to develop accessible and scalable methods that provide more accurate continuous quantitative and visual descriptions of ancestry at multiple resolutions that can replace widely used programs like Admixture. Such developments should be a priority among human geneticists.

The racial hereditarian research discussed also tends to involve datasets where there is often explicit recognition that cross-population comparisons are invalid. The Social Science Genetic Association Consortium explicitly cautions about the invalidity of such analyses and several FAQs associated with GWAS papers acknowledge these limitations as well. Given these cases, it would seem likely that efforts like supplementary FAQs are unlikely to prevent the creation and spread of such flawed analyses. Therefore, approaches should emphasize norms and regulations prior to publishing. For example, the ethical framework for using genetic ancestry laid out in Lewis et al. (2023) ought to be widely adopted by researchers to instill normative commitments to justice, beneficence, and anti-racism alongside truthfulness and to foster careful reflection of research practices tailored to particular study aims. Integrating these values into institutional regulatory structures, like data use certification agreements and manuscript or grant reviews will be crucial. Finally, as Nelson et al. (2022) note, group-level harms like stigmatization are often overlooked in ethical and regulatory frameworks. Incorporating these factors more explicitly in grant reviews, data use certification agreements, and reviews of data access requests might help further prevent misuse of these datasets. Additionally, we urge research consortia and institutional review boards to scrutinize their informed consent processes and ensure that study volunteers and patient populations recruited for genomics research are thoroughly informed about potential group harms that might arise (including through secondary analysis of anonymized data). To prevent the substantial threat of unauthorized data access and distribution that undermines trust and integrity, greater security of datasets and scrutiny of Data Authorization Requests may be required. In a 2020 recommendation, the Secretary’s Advisory Committee on Human Research Protections for the Department of Health and Human Services made a recommendation to adopt “sanctions of sufficient consequence,” including fines, to deter unauthorized use and sharing of data derived from human participants (SACHRP, 2022). They further suggested that sanctions apply whether NIH funding is involved or not and be able to include institutions and investigators regardless of whether they are funded by NIH. We believe this recommendation should be formally adopted into NIH policy and explore what other punitive measures the NIH is capable of levying against researchers outside formal academic affiliations and mainstream scientific communities. Another option includes expanding the role of the US Office of Research Integrity (ORI), the government agency charged with detecting, investigating, and preventing research misconduct. Although the ORI currently uses a very narrow definition of research misconduct (specifically focusing on instances of data falsification, fabrication, and plagiarism), from its inception in 1989 until 2005, the office used a broader definition that also includes “other practices that seriously deviate from those that are commonly accepted within the scientific community for proposing, conducting, or reporting research” (Caron et al., 2023). We propose that this broader definition be readopted by the ORI (and similar institutional units that follow the ORI’s guidelines) to accommodate cases of intentional and egregious misuse or unauthorized access of protected datasets that clearly deviate from the ethical norms within the scientific community.

## Data Availability

The original contributions presented in the study are included in the article/Supplementary material, further inquiries can be directed to the corresponding author.
